# The Level of Methionine Residues in Storage Proteins Is the Main Limiting Factor of Protein-Bound-Methionine Accumulation in Arabidopsis Seeds

**DOI:** 10.3389/fpls.2020.01136

**Published:** 2020-08-05

**Authors:** Aiswarya Girija, David Shotan, Yael Hacham, Rachel Amir

**Affiliations:** ^1^ Department of Plant Science, MIGAL—Galilee Research Center, Kiryat Shmona, Israel; ^2^ Department of Biotechnology, Tel-Hai College, Upper Galilee, Israel

**Keywords:** 2S sunflower albumin, cystathionine-γ-synthase, metabolic profiling, metabolism, methionine, seed storage proteins

## Abstract

The low level of methionine, an essential sulfur-containing amino acid, limits the nutritional quality of seeds. Two main factors can control the level of protein-bound methionine: the level of free methionine that limits protein accumulation and the methionine residues inside the storage proteins. To reveal the main limiting factor, we generated transgenic *Arabidopsis thaliana* seed-specific plants expressing the methionine-rich sunflower seed storage (*SSA*) protein (A1/A2). The contents of protein-bound methionine in the water-soluble protein fraction that includes the SSA in A1/A2 were 5.3- and 10.5-fold, respectively, compared to control, an empty vector (EV). This suggests that free methionine can support this accumulation. To elucidate if the level of free methionine could be increased further in the protein-bound methionine, these lines were crossed with previously characterized plants having higher levels of free methionine in seeds (called SSE). The progenies of the crosses (A1S, A2S) exhibited the highest level of protein-bound methionine, but this level did not differ significantly from A2, suggesting that all the methionine residues of A2 were filled with methionine. It also suggests that the content of methionine residues in the storage proteins is the main limiting factor. The results also proposed that the storage proteins can change their content in response to high levels of free methionine or SSA. This was assumed since the water-soluble protein fraction was highest in A1S/A2S as well as in SSE compared to EV and A1/A2. By using these seeds, we also aimed at gaining more knowledge about the link between high free methionine and the levels of metabolites that usually accumulate during abiotic stresses. This putative connection was derived from a previous analysis of SSE. The results of metabolic profiling showed that the levels of 29 and 20 out of the 56 metabolites were significantly higher in SSE and A1, respectively, that had higher level of free methionine, compared A1S/A2S, which had lower free methionine levels. This suggests a strong link between high free methionine and the accumulation of stress-associated metabolites.

## Introduction

The low levels of methionine, an essential sulfur-containing amino acid, limit the nutritional quality of a plant-based diet (Hesse and Hoefgen, 2003; Amir et al., 2012). Methionine, in addition to its role in protein synthesis, plays a vital role in growth and development processes of plants, but also in humans and animals (Amir et al., 2019). To meet the increasing demand for methionine in humans and animal food and feed, four main approaches have been tested previously in both vegetative and seed tissues: i) upregulating key methionine biosynthesis enzymes to elevate the level of free methionine; ii) expressing methionine-rich storage proteins to incorporate more free methionine into the protein fraction; iii) reducing methionine-poor storage proteins inside the plants to support the accumulation of methionine-rich proteins; and iv) silencing methionine catabolic enzymes [reviewed by Galili and Amir (2013) and Amir et al. (2019)]. In the current study, we tested a combination of the first two approaches in order to determine the rate-limiting factor of protein-bound methionine in *Arabidopsis thaliana* seeds that were used as a model plant.

**Figure 1:**
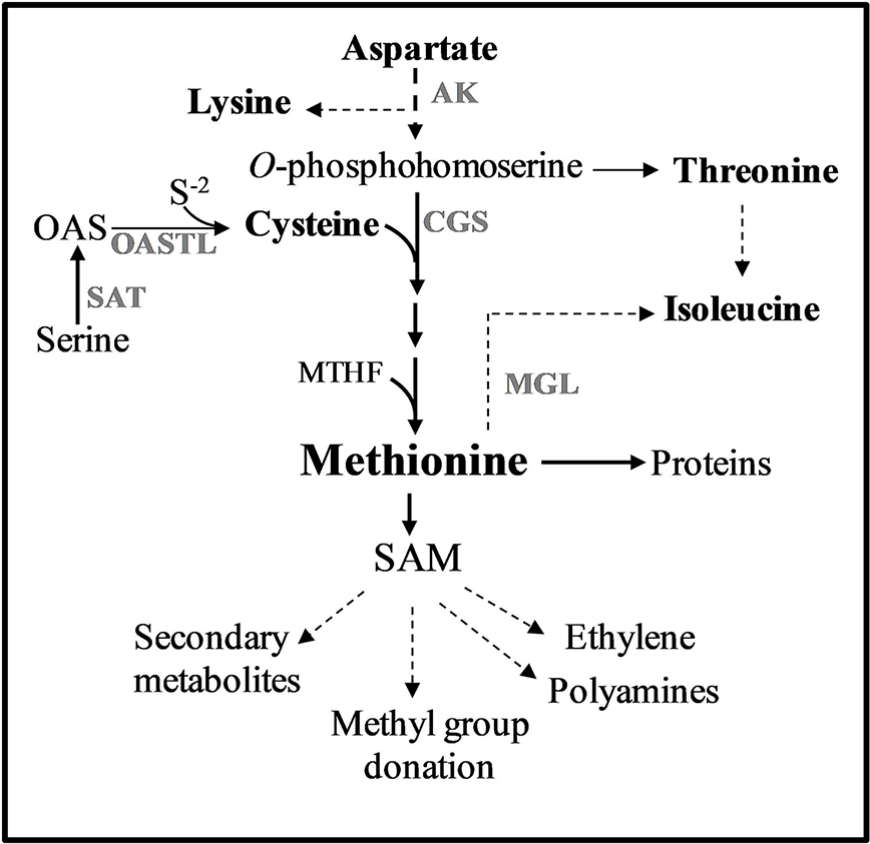
Methionine metabolism in plants. Only some of the enzymes and metabolites are specified. Solid arrows represent one metabolic step while dashed arrows represent multiple metabolic steps. Abbreviations: AK, aspartate kinase; CGS, cystathionine γ-synthase; MGL, methionine γ-lase; SAM, S-adenosyl-methionine; S^-2^, sulfide; OAS, *O*-acetylserine; SAT, serine acetyl transferase; OASTL, *O*-acetylserine thiol lyase.

One of the approaches previously tested to elevate protein-bound methionine was elevating free methionine with the goal of promoting the accumulation of methionine-rich storage proteins. Previous studies have shown that one of the main regulatory enzymes controlling the level of free methionine is its first biosynthesis unique enzyme, cystathionine-γ-synthase (CGS). CGS combines the carbon/amino skeleton from the aspartate pathway with sulfur moiety derived from cysteine (Kim et al., 2002) (**Figure 1**). Indeed, overexpressing the *A. thaliana* *CGS* (*AtCGS*), or seed-specific expression of *AtCGS*, leads to significantly higher levels of free methionine (Kim et al., 2002; Di et al., 2003; Dancs et al., 2008; Hanafy et al., 2013; Song et al., 2013; Cohen et al., 2014; Cohen et al., 2016b). High elevations of free methionine in seeds were also obtained when other genes from the biochemical pathways of the aspartate family and the sulfur assimilation pathway were used (Karchi et al., 1993; Demidov et al., 2003; Kim et al., 2012; Nguyen et al., 2012; Planta et al., 2017; Xiang et al., 2018). In order to achieve a high expression in seeds during the production of proteins, the heterologous genes were fused to the promoters of seed-storage proteins that express the genes in the last stage of seed development when most of the amino acids are formed (e.g., Karchi et al., 1993; Kim et al., 2012; Nguyen et al., 2012; Matityahu et al., 2013; Amir et al., 2018). The highest level of free methionine (sixfold) was detected in the seeds of *A. thaliana* [called SSE plants (Cohen et al., 2014)], expressing the feedback-insensitive form of *AtCGS*, *AtD-CGS* (Hacham et al., 2006).

In addition to high levels of free methionine, the metabolic profile of SSE seeds resembles a profile of plants that suffer from abiotic stress (Cohen et al., 2014). The levels of most of the amino acids, polyols, and sugars increased significantly in these seeds compared to control wild type (WT) seeds. Also, the expression levels of genes associated with abiotic stresses such as early response to dehydration, drought, and osmotic stresses were significantly upregulated in these SSE seeds (Cohen et al., 2014). This phenotype suggests that a high level of methionine causes stress.

The second approach to elevate methionine, which was based on experimental evidence, indicates that although the steady-state level of free methionine is relatively low, the flux towards its degraded metabolites is relatively high. By using radiolabeled isotope in *Lemna punctata* and *A. thaliana*, it was revealed that about 80% and 50% of the methionine was catabolized to its main catabolic product, *S*-adenosyl methionine (SAM) and to its related metabolites, respectively (Giovanelli et al., 1985; Ranocha et al., 2001) (**Figure 1**). SAM is the major methyl donor for methyltransferases and a precursor for various essential metabolites such as ethylene, vitamins, and polyamines (Roje, 2006). Based on the understanding that most of the free methionine drives towards SAM and its associated metabolites, the researchers suggest that increasing the expression level of heterologous genes encoded to methionine-rich seed-storage proteins would compete with that vast degradation and would trap the free methionine into storage proteins. Several methionine-rich storage proteins were used to increase protein-bound methionine. These include the 2S albumin of sunflower (having 16% methionine residues from the total amino acids), 15 kDa zein (having 11%), and the 2S albumin of Brazil nut (having 18%) [reviewed by Tabe et al. (1993) and Galili and Amir (2013)]. Seed-specific expression of these methionine-rich proteins revealed that the levels of protein-bound methionine that incorporates into proteins increased in the transgenic seeds of soybean by 16% (Zhang et al., 2014), 33% in canola (Altenbach et al., 1992), and about 90% in lupin and chickpea seeds (Molvig et al., 1997; Chiaiese et al., 2004). However, in studying the consequences of these manipulations, it was found that these elevations were mostly at the expense of other sulfur-containing metabolites and/or endogenous methionine-rich proteins (Jung, 1997; Molvig et al., 1997; Muntz et al., 1998; Tabe and Droux, 2002; Hagan et al., 2003). Based on these findings, the researchers assume that the low pool of free methionine in seeds limits the accumulation of methionine-rich proteins during grain filling.

In the current study, we aim at i) revealing the main limiting factor that controls the accumulation of protein-bound methionine in *A. thaliana* seeds, which could be either the content of free methionine or the level of methionine residues inside the seed-storage proteins; ii) determining if a combination of increasing methionine biosynthesis with the expression of methionine-rich storage proteins in seeds [also known as the “push–pull” strategy (Tabe et al., 2010)] will lead to higher levels of protein-bound methionine compared to their parents; iii) studying the effect of high methionine levels on the content of water-soluble protein fraction; and iv) gaining more knowledge about the relationship between high free methionine content in seeds and the accumulation of stress-related metabolites.

## Materials and Methods

### Generation of Transgenic *A. thaliana* Plants Expressing Seed-Specific Sunflower Albumin (*SSA*)

The *SSA* gene (GenBank Accession. no: X56686) controlled by the seed-specific vicilin promoter [originating from pea (*Pisum sativum* L.)] and the vicilin terminator in pLT4 plasmid was kindly obtained from Linda Tabe (Molvig et al., 1997). The fragment containing the promoter, the *SSA* gene, and the terminator was then digested from the pLT4 vector using *EcoRI* and introduced into the binary Ti plasmid pGPTV-BAR. This latter plasmid has the *bar* resistance gene for the Basta herbicide. This plasmid was used to transform *Agrobacterium tumefaciens* EHA105 cells. *A. tumefaciens* EHA105 harboring pGPTV-SSA-BAR was used to transform *A. thaliana* Columbia-0 ecotype using the floral dip method. Transformed seeds were selected on medium supplemented with Basta (5 µg ml^-1^). Thirteen independent transformation events were selected and planted in an enriched soil medium. Seeds were collected from the T_1_ generation and then analyzed for a 3:1 segregation based on the presence of the transgene and the observed resistant phenotype to Basta. Segregated T_1_ lines were then regrown, and the T_2_ generation was examined for non-segregating lines to produce T_2_ and further T_3_ homozygous lines. Two T_3_ homozygous lines designated as A1 and A2, showing the highest levels of SSA protein accumulation by immunoblot analysis, were chosen for further analysis.

### Generation of Transgenic *A. thaliana* Plants Co-expressing the Seed-Specific *SSA* and the Feedback-Insensitive Form of Cystathionine-γ-Synthase *(AtD-CGS)*

The selected homozygous transgenic plants (A1 & A2) were crossed with the fifth generation of SSE lines expressing the seed-specific *AtD-CGS* (Cohen et al., 2014).* *For the crossing events, the unopened flowers were hand-pollinated and the seeds were harvested after maturation. A plant having an empty vector (EV) was used as the experimental control (Cohen and Amir, 2017). The progenies resulted from the independent crosses, A1 x SSE and A2 x SSE, referred to as A1S and A2S, were grown on a 0.5% Murashige and Skoog (MS) medium (Duchefa) containing 0.5% sucrose and 7 mg ml^-1^ plant Agar (Duchefa). T_1_ and T_2_ lines of seeds expressing *SSA* alone (A1, A2) and crosses (A1S, A2S) were selected on media supplemented with 5 µg ml^-1^ Basta. Since the SSE seeds lost their kanamycin resistance during the generation, the presence of *AtD-CGS* gene was confirmed by PCR analysis. Genomic DNA was extracted from the leaves of 30 independent progenies of each A1S and A2S line and was screened by PCR using gene-specific primers (**Supplementary Table S1**). Seedlings were transferred to fertilized soil after 12 days of germination and grown at 22 ± 2°C under a 16 h/8 h light/dark photoperiod. T_3_ generations of all genotypes including the control EV plants were grown at the same time in an environmentally controlled growth chamber. Mature dry seeds were harvested followed by drying and were stored at 4°C.

### Quantitative Real-Time PCR and Immunoblot Analyses

For transcript determination, total RNA was extracted from 50 mg of mature dry seeds using the Spectrum Plant Total RNA kit (Sigma). One microgram of RNA was used for cDNA biosynthesis using the Verso cDNA biosynthesis kit (Thermo Scientific) according to the manufacturer’s protocol. The expression of mRNA levels of *AtD-CGS* and *SSA* in EV and transgenic plants was quantified using quantitative real-time PCR (qRT-PCR). To normalize variance among samples, protein phosphatase 2A subunit A3 (*AtPP2A-A3*) transcript level was used as an endogenous control (Cohen et al., 2014). The values presented are means of five biological replicates. All primers used for qRT-PCR analyses are listed in **Supplementary Table S1**.

For the immunoblot analysis, water-soluble protein fraction was extracted from 20 mg dry seeds of EV and transgenic seeds, A1, A2, A1S and A2S, as previously described (Matityahu et al., 2013). The seeds were ground in 400 μl of 25 mM Na phosphate buffer pH = 7.8 with a protease inhibitor cocktail (Sigma, P9599). After two centrifugation cycles (20,000 g at 4°C for 20 min), the upper phase was collected and 20 μl was loaded onto the gel. The expression level of the *SSA* (6.5 kDa) protein was detected with *SSA* anti-serum that was kindly obtained from Rainer Hoefgen (Max Planck Institute of Molecular Plant Physiology, Germany).

### Primary Metabolic Profiling, Free and Protein-Bound Amino Acids Determination Using GC-MS

Mature dry seeds from different transgenic sets were collected separately from eight distinct plants. Twenty milligrams of mature dry seeds from control and transgenic lines were ground into a fine powder using liquid nitrogen and a Restch MM 301 homogenizer, as previously described (Cohen et al., 2014; Cohen et al., 2016b). The crude extract was separated into polar and non-polar phases with methanol/water/chloroform. The upper polar phase was vacuum-dried and dissolved in 40 µl of 20 mg ml^-1^ methoxyamine hydrochloride in pyridine for 2 h, followed by derivatization for 30 min in N-methyl-N(trimethylsilyl)-trifluoroacetamide at 37°C for 2 h with vigorous shaking. One microliter of sample was injected into a GC-MS system with a split ratio of 1:1, together with the amino acid standards of 5, 10, 25, 50, 100 and 200 µM. For the free amino acid, the single-ion mass method was used with the RXI-5-Sil MS capillary column (RESTEK; 30 m, 0.25-mm i.d., and 0.25-mm thickness), while the total-ion-count method was used for metabolic profiling and separation using the VF-5ms capillary column (Agilent; 30 m + 10 m EZ-guard, 0.25-mm i.d., and 0.25-mm thicknesses). All analyses were carried out on a GC-MS system (Agilent 7890A) coupled with a mass selective detector (Agilent 5975c) and a Gerstel multipurpose sampler (MPS2; Cohen et al., 2014). Norleucine (2 mg ml^-1^ in HPLC grade water) and Ribitol (2 mg ml^-1^ in HPLC grade water) were used as internal standards. The peak areas were calculated from the standard calibration curves and normalized to the internal controls (norleucine and ribitol) signal.

For the analysis of water-soluble protein-bound amino acid content, 20 mg of seeds was ground into powder using a Restch MM 301 homogenizer suspended in 400 µl of water containing 2 mg ml^-1^ norleucine and vigorously vortexed. After two centrifugation cycles (20,000 g at 4°C for 20 min), the water-soluble protein fraction was determined from the upper phase. Forty microliter of water-soluble protein was dried under vacuum and sent for protein hydrolysis (Department of Chemical Research Support, Weizmann Institute of Science, Rehovot, Israel). Samples of the hydrolyzed proteins were separated using 300 µl of water and 300 µl of chloroform. From the aqueous phase, 200 µl of the sample was dried under vacuum and derivatized, as described for the free amino acid analysis. The data were collected from five biological replicates and analyzed according to the standard curve for each amino acid.

### Quantification of the Content of the Water-Soluble Proteins

To quantify the water-soluble protein, 20 mg of seeds was ground into powder using a Restch MM 301 homogenizer suspended in 400 µl of 25 mM Na phosphate buffer pH = 7.8 and vigorously vortexed. After two centrifugation cycles (20,000 g at 4°C for 20 min), the water-soluble protein fraction was determined from the upper phase using a Bradford reagent. Bovine serum albumin was used as a standard.

### Determination of Seed Weight and Germination Assay

The dry seed weight was determined from exactly 100 seeds of each genotype in four biological replicates. For the germination assay, 30 seeds from each of these genotypes in five replicates were placed on the same day in a petri dish having 0.5% MS medium (Duchefa), supplemented with 0.5% sucrose (J.D. Baker) and 7 mg ml^-1^ plant agar (Duchefa). After 48 h in a cold room (4°C), the plates were transferred to a growth room (22 ± 2°C, 16/8-h light/dark cycle at 100 μmol m^–2^ s^-1^). The rate of germination was determined by observing the emergence of radicals 2 days post-transferring to the growth room.

### Statistical Analysis

The normalized data were used for multivariate statistical analysis, including principal component analysis (PCA). Heat maps were used to visualize metabolite responses and were generated based on the average of each metabolite in five biological replicates. PCAs and heat maps were done using the Metaboanalyst 4.0 software (https://www.metaboanalyst.ca/; Chong et al., 2018). The graphs for the experiments were performed using the GraphPad Prism 8 scientific software. Student’s t-test and analysis of variance (ANOVA) were done using the JMP 8 software: a P value of <0.05 was considered statistically significant for the experiments.

## Results

### Seeds of Transgenic *A. thaliana* Plants Expressing a Methionine-Rich Sunflower Albumin Had a Higher Level of Protein-Bound Methionine

**Figure 2:**
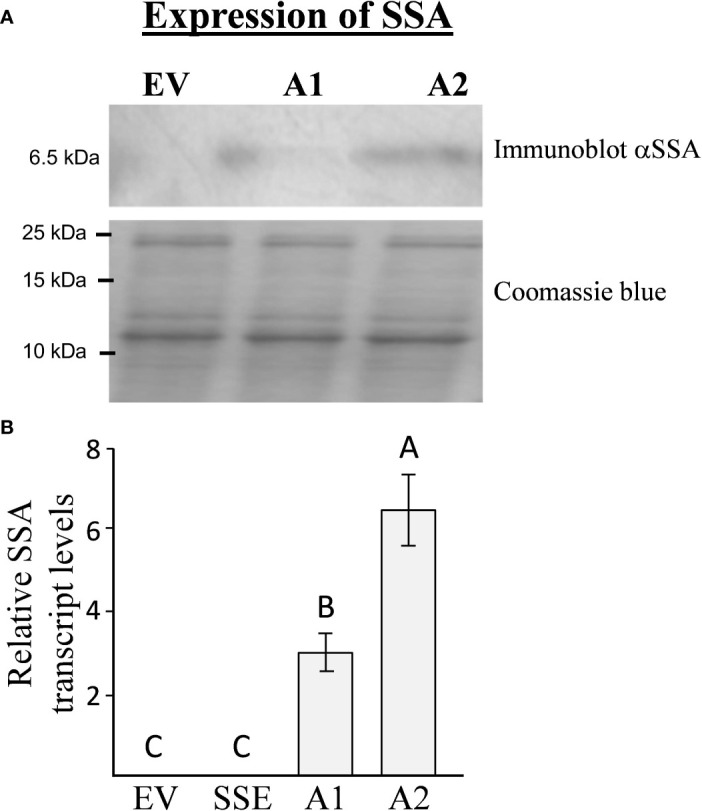
The expression levels of *SSA* in transgenic seeds**. (A)** Immunoblot analysis of *SSA* in A1/A2 transgenic seeds expressing this gene. The upper panel represents the signal using antibodies against the *SSA* protein, while the bottom panel represents crude protein extracts counterstained with coomassie brilliant blue used for equal loading. The marker size is shown on the left. **(B)** qRT-PCR analysis of *SSA* transcript levels in lines expressing the *SSA* gene (A1, A2) and in the empty vector (EV) line used as a control. The results are normalized according to the expression of constitutive gene *AtPP2A-A3*. The data are presented as the mean ± SD of five individual plants per line. Statistically significant changes (Tukey-Kramer HSD test, *P* ≤ 0.05) between plants are identified by different letters.

To introduce the “push–pull” strategy in seeds, we generated *A. thaliana* plants expressing the methionine-rich 6.5 kDa 2S albumin from sunflower (*SSA*) under the control of the seed-specific vicilin promoter. The seeds of 13 independent Basta-resistant transgenic lines were screened for their SSA protein content using immunoblot analysis. Two homozygous lines designated as A1 and A2, having the highest expression levels of SSA, were chosen for further study (**Figure 2A**). Quantitative real-time PCR (qRT-PCR) analyses showed that A2 has 2.1-fold higher expression levels of the transgene *SSA* compared to A1 (**Figure 2B**), which is in accordance with the results obtained from the immunoblot analysis (**Figure 2A**). Plants having an empty vector (EV) were used as a control for this study (Cohen and Amir, 2017).

To determine if a high level of SSA would lead to increased incorporation of free methionine into the water-soluble protein fraction, which also included the albumin fraction, this protein fraction was extracted from A1/A2 seeds. This fraction was subjected to protein hydrolysis, and the level of methionine was measured. The results showed that the levels of protein-bound methionine in this fraction had increased significantly up to 5.3- and 10.5-fold in the seeds of A1 and A2, respectively, compared to the control EV seeds (**Table 1**). The accumulation of protein-bound methionine in A1/A2 seeds correlated well with the expression levels of SSA both at the transcript and protein levels (**Figure 2**).

**Table 1:** The level of protein-bound amino acids in dry seeds expressing *AtD-CGS* (SSE), *SSA* (A1, A2), those expressing the two genes (A1S, A2S), and the control EV after protein hydrolysis^#^.

	EV	SSE	A1	A2	A1S	A2S
Alanine	8.01 ± 1.60 a	12.53 ± 2.82 a	11.32 ± 1.61 a	13.23 ± 2.38 a	14.24 ± 4.34 a	14.88 ± 4.70 a
Valine	5.76 ± 1.43 a	8.46 ± 1.74 a	8.80 ± 1.99 a	8.36 ± 1.29 a	6.19 ± 1.53 a	7.13 ± 1.84 a
Serine	4.38 ± 0.93 b	6.80 ± 1.33 b	7.00 ± 0.96 b	8.88 ± 1.47 a	7.927 ± 2.199 b	8.98 ± 2.77 a
Leucine	5.30 ± 0.89 c	9.41 ± 2.00 bc	11.00 ± 1.71 bc	17.32 ± 3.24 a	15.32 ± 4.64 b	17.35 ± 5.04 a
Threonine	4.70 ± 0.96 a	7.72 ± 1.59 a	6.43 ± 0.75 a	8.17 ± 1.57 a	7.76 ± 2.35 a	8.56 ± 2.54 a
Isoleucine	2.99 ± 0.50 b	4.63 ± 0.86 b	4.68 ± 0.57 b	5.90 ± 0.95 a	5.48 ± 1.85 ab	6.24 ± 1.73 a
Proline	6.89 ± 2.23 a	7.92 ± 1.57 a	10.75 ± 2.27 a	10.13 ± 1.65 a	9.44 ± 2.32 a	10.20 ± 3.19 a
Glycine	3.45 ± 0.17 c	6.28 ± 1.39 bc	6.66 ± 0.68 a	11.72 ± 1.50 b	9.29 ± 5.00 bc	13.78 ± 4.33 a
Methionine	0.75 ± 0.16 c	1.46 ± 0.46 b	3.99 ± 0.20 b	7.93 ± 1.14 a	7.82 ± 0.95 a	8.71 ± 2.01 a
Aspartate	28.46 ± 12.84 a	39.56 ± 9.26 a	42.60 ± 7.26 a	56.81 ± 8.22 a	41.30 ± 25.69 a	54.20 ± 19.19 a
Phenylalanine	2.87 ± 0.47 b	5.01 ± 1.09 b	4.26 ± 0.60 b	5.95 ± 1.23 a	5.40 ± 1.81 b	6.50 ± 2.42 a
Glutamate	27.71 ± 7.37 b	46.51 ± 9.74 b	49.13 ± 7.69 b	73.39 ± 13.42 a	63.97 ± 19.99 a	79.72 ± 29.88 a
Lysine	13.06 ± 3.07 b	27.70 ± 6.80 b	25.27 ± 6.14 b	32.45 ± 4.98 a	23.37 ± 9.60 b	32.91 ± 11.60 a
Tyrosine	1.35 ± 0.38 b	2.79 ± 0.63 b	2.78 ± 0.41 b	4.01 ± 0.65 a	2.74 ± 1.19 b	4.07 ± 1.84 a
Total AA’s	115.71 ± 32.67 b	186.81 ± 39.05 ab	194.71 ± 29.65 ab	264.31 ± 42.26 a	219.13 ± 63.77ab	273.25 ± 93.12 a

^#^The levels of amino acids after protein hydrolysis. The quantities of amino acids were analyzed using GC-MS, and their levels were normalized to norleucine and calculated as μmol per g dry weight of seeds. Values are the mean ± standard deviation of five biological replicates of 20 mg seeds isolated from five plants per line. Significance between plants was calculated according to the Turkey-Kramer HSD test (p < 0.05) and is identified by different letters.

### Seeds of the Transgenic Plants Showed Differences in Their *AtD-CGS* and *SSA* Expression Levels Compared to EV

With the aim of understanding whether the levels of free methionine are a limiting factor in the accumulation of methionine in the storage proteins of A1/A2 seeds, and if so, whether the methionine level could be increased further in these seeds, we crossed between the homozygous lines expressing *SSA* with the fifth generation of homozygous SSE plants. This latter plant expressed *AtD-CGS* under the control of the seed-specific phaseolin promoter and had a high level of free methionine (Cohen et al., 2014). Two independent crossing events were carried out: A1 x SSE (termed A1S) and A2 x SSE (termed A2S). Since SSE lines lost their kanamycin resistance after several generations (but they had the NptII gene and the *AtD-CGS*), the progenies of these crosses were germinated on half-MS media with Basta, and 15 Basta-resistant plants from each line of the crosses were screened by PCR for the presence of both genes, *SSA* and *AtD-CGS*. The seeds from T_3_ homozygous progenies were used for further analysis.

**Figure 3:**
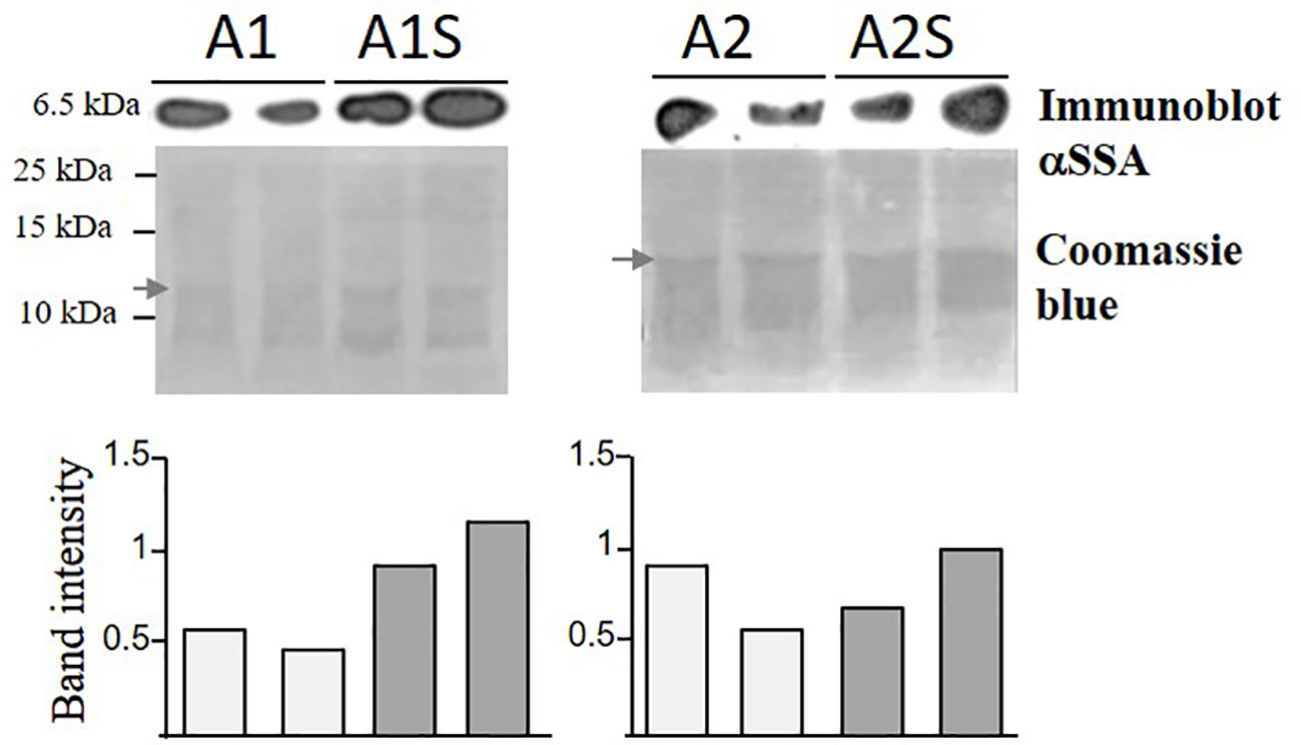
Immunoblot analysis of *SSA* in transgenic seeds expressing *SSA* alone (A1, A2) and in seeds expressing *SSA* and *AtD-CGS* (A1S, A2S). For the analysis, water-soluble proteins were extracted from an equal amount of 20 mg seeds from each sample with the addition of an equal amount of extraction buffer. Two lines for each genotype were tested. Upper panels: immunoblot analysis using antibodies against the protein of *SSA*. Lower panel: coomassie brilliant blue staining used for equal loading. The marker size is shown on the left. The graph represents band intensity of the SSA normalized to a band from coomassie (marked by an arrow) as measured by ImageJ. The two gels were run separately.

The transcript expression levels of the *SSA* and *AtD-CGS* in the parents and those expressing the two heterologous genes were measured using quantitative real-time PCR (qRT-PCR) (for details, see **Supplementary Figure S1**; **Supplementary Text**). To reveal if the level of free methionine limits the accumulation of *SSA*, an immunoblot analysis was carried out using anti-*SSA* antibodies on the water-soluble protein fraction. If that limitation exists, the protein level of *SSA* is expected to increase in the progenies of the crosses (A1S, A2S) above the level of their parents, A1 and A2. The results showed that the *SSA* level was higher in two lines of A1S (by about 50–100%) than in its parent, A1, but such an elevation was not observed in A2S, which exhibited a level of *SSA* similar to its parent, A2 (**Figure 3**). In addition, coomassie-brilliant blue staining showed a higher level of other water-soluble storage proteins in A1S, compared to A1 (**Figure 3**). These results indicated that the seed-specific expression of *AtD-CGS* led at least in A1S to higher levels of *SSA*, which in turn also supported the accumulation of other storage proteins in this line. The observation that A2S had a level of *SSA* similar to A2 (**Figure 3**) indicates that the capacity for methionine residues was fulfilled in the A2 line (which has a twofold higher expression level of *SSA* compared to A1), and thus, a higher level of free methionine (caused by the expression of *AtD-CGS*) failed to raise the level of *SSA* significantly beyond that detected in A2 in the water-soluble protein fraction.

**Figure 4:**
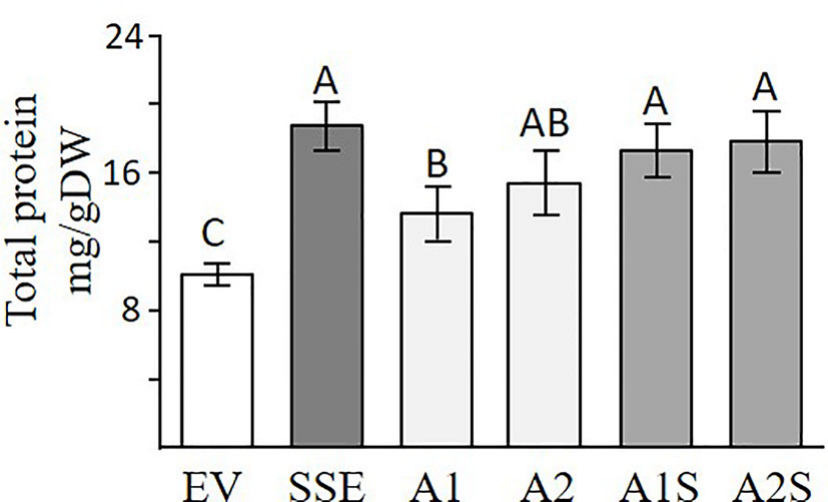
Total water-soluble protein fractions analyzed by Bradford assay. Water-soluble proteins fractions in seeds expressing the *SSA* gene (A1, A2), in seeds expressing *SSA* and *AtD-CGS* (A1S, A2S), and in SSE and EV. The assay was done on extracts from 20 mg seeds/dry weight. The data are presented as the mean ± SD from four individual plants. Statistically significant changes (Tukey-Kramer HSD test, *P* ≤ 0.05) between plants are identified by different letters.

A Bradford assay was used to determine if a higher expression of SSA affects the total water-soluble protein fraction. This fraction (which also includes SSA) is the second major fraction after the 12S globulins (cruciferin), and it accounts for about 20% of the total proteins in seeds of *A. thaliana* (Fujiwara et al., 2002). The results showed that the seeds of A1 and A2 had 24% and 38% higher levels of water-soluble protein, respectively, compared to EV (**Figure 4**), suggesting that the expression of SSA can increase the level of this fraction. The results also demonstrated that the SSE seeds had the highest water-soluble protein abundance of 69% compared to EV, while those of A1S and A2S had 51% and 58% higher levels, respectively, compared to EV, which did not differ significantly from SSE (**Figure 4**). This suggests that the co-expression of *AtD-CGS* and *SSA* in *A. thaliana* seeds does not elevate the level of water-soluble proteins fraction beyond the level detected in SSE seeds expressing *AtD-CGS* alone. Together, the results showed that a combination of *SSA* and *AtD-CGS* led to higher water-soluble protein levels in A1S compared to A1, while A2S had an insignificant elevation of 16% compared to A2.

### Effect of Co-expression of *AtD-CGS* and *SSA* on the Levels of Free and Protein-Bound Methionine, and on the Other Amino Acids in the Transgenic Seeds

**Figure 5:**
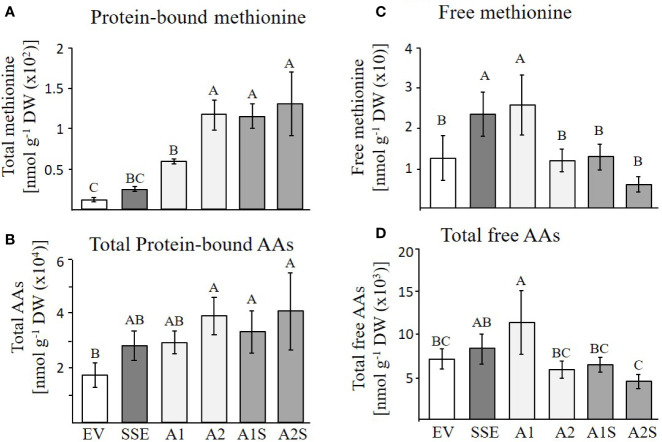
GC-MS analyses of free and protein-bound methionine and other amino acids. **(A, B)** Protein-bound levels of methionine and total amino acids in the transgenic dry seeds of A1S, A2S, A1, A2, SSE, and EV followed by protein hydrolysis; **(C, D)** Free levels of methionine and total amino acids in the same set of seeds. Data shown are means ± SD of five biological replicates. Statistically significant changes (Tukey-Kramer HSD test, *P* ≤0.05) between plants are identified by different letters.

Based on the accumulation of SSA in the progenies of A1S (**Figure 3**), and the high level of the water-soluble protein fraction in the seeds of A1S/A2S, we assume that most of the free methionine in these lines was incorporated into SSA, and thus, the levels of protein-bound methionine would increase. To test these assumptions, the water-soluble protein fraction was isolated from the seeds of the different lines and was used for protein hydrolysis followed by GC-MS analysis. The results showed that the level of protein-bound methionine increased significantly in A1S/A2S (**Figure 5A**). By comparing the level of protein-bound methionine in the seeds of the progenies of the crosses lines with their parents A1/A2, it was found that proteinbound methionine increased significantly by 96% in A1S compared to A1, but in A2S, the elevation (10%) was insignificant compared to A2 (**Figure 5A**). The SSE seeds showed a twofold increase in the level of protein-bound methionine compared to EV (**Figure 5A**), which is consistent with the previous report showing a 1.6-fold increase in the levels of protein-bound methionine of the SSE seeds (Cohen et al., 2014). In comparison to the SSE seeds, the levels of protein-bound methionine contents in the A1S and A2S seeds increased 5.4- and 5.9-fold, respectively, and compared to EV, their levels increased 10.4- and 11.5-fold, respectively (**Figure 5A**; **Table 1**). Together, the results showed that the level of protein-bound methionine increased significantly in A1S compared to A1, suggesting a potential of the “pull–push” approach. However, the results obtained from A2S and A2 suggest that they reach their maximum capacity to incorporate free methionine, and that the methionine residues in the storage proteins become the main limiting factor of the protein-bound methionine content.

The higher levels of water-soluble proteins in the A1S and A2S transgenic lines (**Figure 4**) and the previous findings that higher levels of free methionine in SSE affect the levels of other amino acids (free and protein-bound) (Cohen et al., 2014) suggest a possible elevation of other amino acids in the water-soluble protein fraction in the progenies of the crosses. Indeed, our results showed that the water-soluble protein bound amino acids were significantly higher in A2, A1S, and A2S compared to A1, SSE, and EV. Compared to EV, the accumulation of protein-bound amino acids was highest in A2S (2.36-fold), while A1S showed an elevation of only 89%. The level in A2, however, was not significantly altered compared to A2S and had a 2.28-fold higher level compared to EV (**Figure 5B**; **Table 1**). The levels in SSE and A1 were 61% and 68% higher compared to EV. The amino acids that showed the highest elevations in A2 and A2S were leucine, serine, isoleucine, lysine, glutamate, phenylalanine, and tyrosine, while the levels of most of the other amino acids did not differ significantly between the different sets of seeds (**Table 1**). The results showed that a higher expression level of *AtD-CGS* and/or *SSA* increased the total levels of other amino acids in addition to methionine.

In *A. thaliana* seeds, about 7% of the amino acids were found in their free form (Cohen et al., 2014). Therefore, we next studied how their levels in the different transgenic lines with the assumption that their levels would decrease compared to the SSE seeds. To test this assumption, the levels of free methionine and other amino acids were determined using GC-MS. In all the transgenic lines A2, A1S, and A2S, the level of free methionine was significantly reduced compared to EV. As expected from our previous study (Cohen et al., 2014), the level of free methionine in SSE increased 1.8-fold, while in A1S and A2, the levels were similar to those detected in EV, and A2S showed a reduction of about twofold compared to EV. Notably, compared to SSE, the seeds of A1S and A2S showed a significant 1.7- and 3.6-fold reduction in free methionine content, respectively (**Figure 5C**), suggesting that most of the free methionine incorporated into the seed proteins and mostly to SSA. However, A1 showed an approximately twofold higher free methionine level compared to EV, which is similar to that detected in SSE (**Figure 5C**; **Table 2**).

**Table 2:** The level of free amino acids in dry seeds expressing *AtD-CGS* (SSE), *SSA* (A1, A2), those expressing the two genes (A1S, A2S), and the control EV^#^.

	EV	SSE	A1	A2	A1S	A2S
Alanine	0.26 ± 0.24 c	0.52 ± 0.16 a	0.53 ± 0.11 a	0.30 ± 0.02 bc	0.56 ± 0.07 a	0.47 ± 0.08 ab
Valine	1.02 ± 0.18 b	1.58 ± 0.44 b	2.38 ± 1.10 a	1.16 ± 0.30 b	1.25 ± 0.19 b	0.98 ± 0.17 b
Serine	1.76 ± 0.06 ab	1.81 ± 0.39 ab	2.23 ± 0.54 a	1.42 ± 0.16 bc	1.31 ± 0.17 c	1.07 ± .0.18 c
Leucine	0.34 ± 0.04 bc	0.58 ± 0.25 b	0.77 ± 0.37 a	0.28 ± 0.11 c	0.33 ± 0.05 c	0.22 ± 0.06 c
Threonine	1.08 ± 0.18 a	0.97 ± 0.19a	1.09 ± 0.17 a	0.67 ± 0.12 b	0.95 ± 0.14 a	0.55 ± 0.09 c
Isoleucine	0.44 ± 0.02 c	0.79 ± 0.38 b	1.18 ± 0.59 a	0.45 ± 0.16 c	0.43 ± 0.06 c	0.32 ± 0.07 c
Proline	0.24 ± 0.11 b	0.33 ± 0.24 ab	0.56 ± 0.40 a	0.14 ± 0.05 b	0.10 ± 0.02 c	0.10 ± 0.03 c
Glycine	0.19 ± 0.03 c	0.29 ± 0.15 b	0.35 ± 0.14 a	0.20 ± 0.08 c	0.18 ± 0.03 c	0.17 ± 0.07 c
Homoserine	0.10 ± 0.04 b	0.19 ± 0.16 ab	0.25 ± 0.07 a	0.12 ± 0.04 ab	0.14 ± 0.04 ab	0.07 ± 0.01 b
Methionine	0.12 ± 0.06 b	0.23 ± 0.06 a	0.26 ± 0.09 a	0.12 ± 0.03 b	0.13 ± 0.04 b	0.06 ± 0.01 b
Aspartate	0.54 ± 0.25 ab	0.51 ± 0.14 b	0.82 ± 0.27 a	0.48 ± 0.06 b	0.47 ± 0.12 b	0.19 ± 0.04 c
Phenylalanine	0.27 ± 0.17 ab	0.22 ± 0.07 ab	0.28 ± 0.11 a	0.13 ± 0.02 bc	0.22 ± 0.05 ab	0.03 ± 0.01 c
Glutamate	0.47 ± 0.31 a	0.21 ± 0.09 a	0.36 ± 0.23 a	0.13 ± 0.04 ab	0.05 ± 0.03 c	0.02 ± 0.01 c
Tyrosine	0.07 ± 0.05 c	0.75 ± 0.4 bc	0.14 ± 0.03 ab	0.08 ± 0.02 bc	0.14 ± 0.04 a	0.07 ± 0.02 c
Tryptophan	0.12 ± 0.05 b	0.11 ± 0.06 b	0.21 ± 0.10 a	0.08 ± 0.01 bc	0.06 ± 0.01 c	0.03 ± 0.01 d
Total AA’s	7.08 ± 1.25 bc	8.45 ± 1.98 b	11.45 ± 3.84 a	5.82 ± 1.10 bc	6.36 ± 0.89 bc	4.42 ± 0.71 c

^#^The quantities of free amino acids were analyzed using GC-MS, and their levels were normalized to norleucine and calculated as μmol per g dry weight of seeds. Values are the mean ± standard deviation of five biological repetitions of 20 mg seeds isolated from five plants per line. Significance between plants was calculated according to the Turkey-Kramer HSD test (p < 0.05) and is identified by different letters.

Next, the effects of these manipulations were tested on the levels of the other free amino acids. A similar effect of reduction was observed in A2, A1S, and A2S. The levels of total free amino acids were 19% higher in SSE compared to EV, but were 11% and 60% lower in the progenies of the crosses A1S and A2S, respectively (**Figure 5D**). Compared to EV, A1 had 62% higher content of total free amino acids, but A2 showed a 21% lower level. Furthermore, the low content of free amino acids in A2, A1S, and A2S (**Figure 5D**) compared to SSE strongly suggests that these amino acids incorporated into *SSA* and other seed proteins (**Figure 5B**).

Taken together, the results showed that the contents of protein-bound methionine and other amino acids increased significantly in A1S, A2S, and A2. This shows that a higher level of methionine in A2 does not support the further accumulation of methionine, where apparently most of the methionine residues in the proteins of this line were filled with methionine, which is unlike A1. The levels of free methionine and other free amino acids in A2 and A1S were similar to EV, and in A2S, it decreased to about twofold compared to EV, suggesting that most of the free amino acids incorporated into the storage proteins.

### Evidence of Metabolic Changes in the Different Sets of Transgenic Seeds

**Figure 6:**
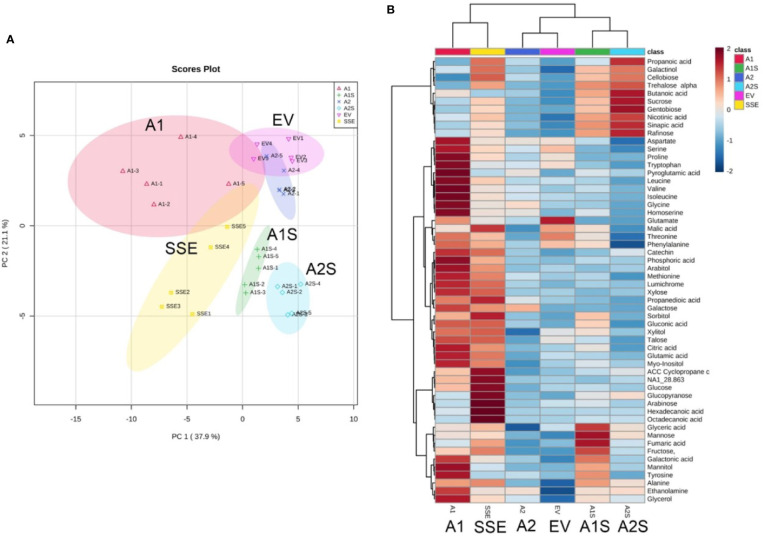
Graphical representation of changes in metabolite profiles in the transgenic seeds. **(A)** Principal component analysis (PCA) according to their entire primary metabolome set of 56 metabolites measured by GC-MS analysis. The data points are displayed as projections onto the two primary axes (eigenvectors). Variances explained by the first two components (PC1 and PC2) appear in parentheses. **(B)** Schematic representation of these metabolites in a heat map. The intensity of blue or red represents the value of the coefficient, as indicated on the color scale. The data are represented as the average value of five biological replicates.

Changes in methionine and amino acid contents can affect the accumulation of other metabolites in plants (Galili et al., 2016; Amir et al., 2019). Having seeds that co-express *AtD-CGS* and *SSA* with a lower level of free methionine than that detected in SSE can enable us to examine our fourth goal, which is to reveal the link between high free methionine and the metabolic stress phenotype. The presence of such a link was assumed from measurements carried out in *A. thaliana* and soybean seeds expressing the *AtD-CGS* (Song et al., 2013; Cohen et al., 2014; Cohen et al., 2016b). These seeds had higher levels of free methionine, and their primary metabolic profiles resembled plants suffering from abiotic stress with high levels of amino acids, polyols, and sugars. These seeds also showed up-regulation of genes related to abiotic stress (Cohen et al., 2014; Cohen and Amir, 2017). We hypothesized that this phenotype was due to the presence of high levels of free methionine in these transgenic seeds. If this is the case, then in the seeds of A1S/A2S where the levels of free methionine were reduced compared to SSE and A1, the levels of stress-related metabolites are expected to decrease. To examine this assumption, global primary metabolic profiling was performed on the seeds of the different transgenic lines. A total of 56 metabolites were detected by GC-MS, and the metabolic overview of the seeds was plotted onto a principal component analysis (PCA) to visualize the relationship between the samples (**Figure 6A**). The PCA analysis showed that A1 and A2 were separated from the A1S and A2S crosses lines; that SSE and A1, which had higher levels of free methionine, were relatively closed; and that A2 and EV were also relatively closed (**Figure 6A**; **Supplementary Figure S2**).

The heat-map analysis showed that most of the metabolites were higher in SSE and A1, which had higher levels of free methionine compared to other sets of seeds (**Figure 6B**). Twenty-nine metabolites showed relatively higher levels in SSE compared to A1S and A2S: sugars (arabinose, glucose, fructose, galactose, glucopyranose, xylose, talose, and cellobiose); sugar acids and organic acids (citric, malic, propanedioic, gluconic, hexadecanoic, and octadecanoic acids); amino acids (glutamate, serine, leucine, isoleucine, threonine, tryptophan, glycine, and proline); and polyols (myo-inisitol, xylitol, sorbitol, mannitol, and arabitol) (**Table 2**; **Supplementary Table S2**). Moreover, the level of catechin, which associated with abiotic stress responses, was significantly reduced in A1S and A2S compared to SSE (**Supplementary Table S2**). In addition, 20 metabolites increased significantly in A1 compared to the other lines where 18 metabolites overlapped with SSE. Compered to SSE, A1 had significantly higher levels of valine, phosphoric acid, and aspartate (**Table 2**; **Supplementary Table S2**).

To determine if these low levels of metabolites are correlated to methionine, a correlation analysis was made of all transgenic sets. All of the top 25 metabolites that correlated to methionine had a positive correlation (**Supplementary Figure S3**): the amino acids leucine, isoleucine, valine, serine, homoserine aspartate, glycine, proline, tryptophan, threonine, and phenylalanine; the sugars xylose, galactose, talose, and glucose; the organic acids citrate, phosphoric acid, propanedioate, pyroglutamate, and gluconate; and the polyols sorbitol, myo-inisitol, arabitol and mannitol, and catechin.

In order to present an overview map of the correlations, to detect other metabolites that correlated to methionine, and to explore the metabolites that were negatively correlated to methionine, a Pearson’s pairwise correlation coefficients analysis for each pair of the 56 different metabolites was performed (**Supplementary Figure S4**). This analysis showed that 43 metabolites were positively correlated to methionine, and only 13 metabolites were negatively correlated to methionine: mannose, raffinose, sucrose, gentobiose, trehalose, cellobiose, glycerate, fumarate, butanoic acid, nicotinic acid, sinapic acid, fructose, and galactinol (**Supplementary Figure S4**). Notably, all of these 13 metabolites showed higher levels in A1S and A2S compared to A2 and EV, while the other metabolites were similar to those detected in A2 and EV (**Figure 6B**; **Supplementary Table S2**).

Together, the results from metabolic profiling showed that most of the annotated metabolites decreased significantly in crosses lines that have low levels of free methionine compared to SSE and A1 seeds that had higher levels of free methionine. Our results strengthen the link between high free methionine and the accumulation of metabolites related to stress.

### Seed Weight and Germination Rate of Transgenic Seeds

**Figure 7:**
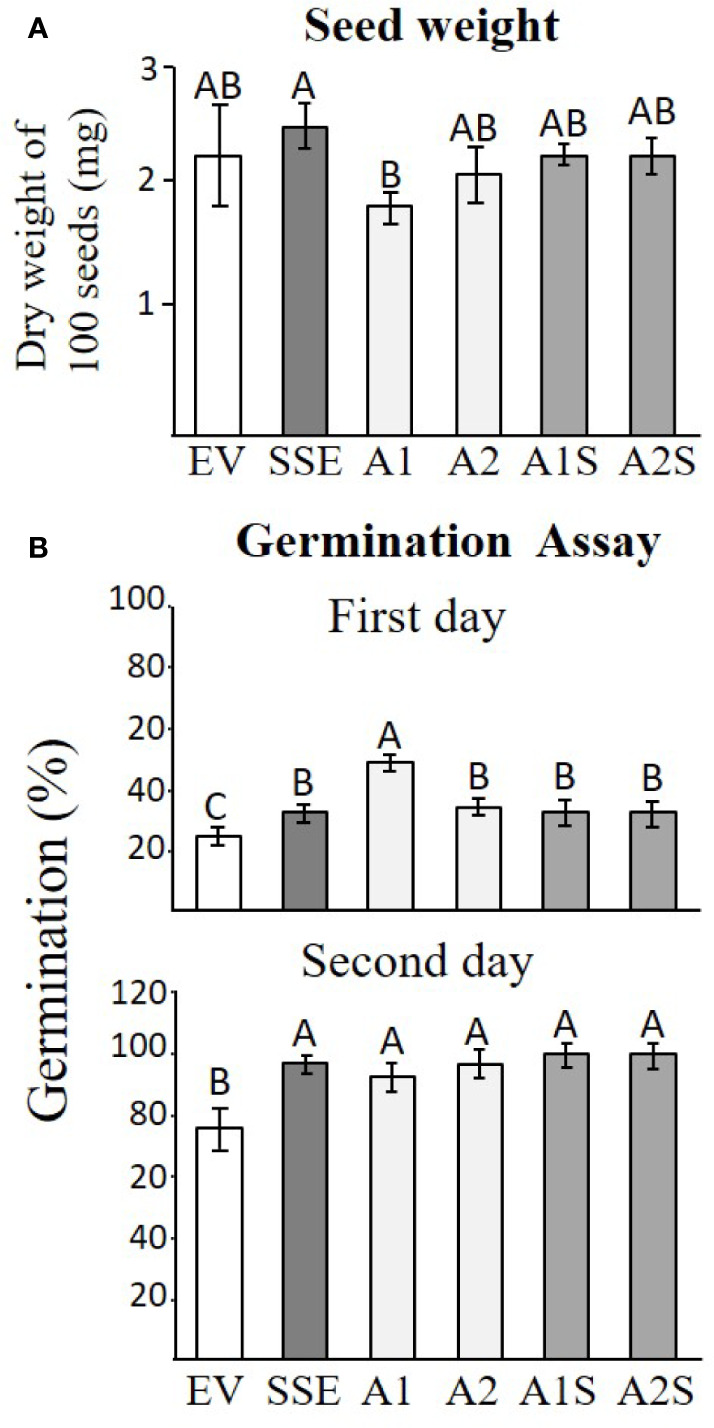
Seed weight and germination efficiency. **(A)** The seed weight of 100 seeds of the different transgenic lines of EV, SSE, A1, A2, A1S, and A2S. Data shown are means of four biological replicates. **(B)** The germination rate on the first and second days after the plates were placed for 2 days in a cold room and were then transferred to a culture room. The rate of germination was determined by observing the emergence of radicals. Data shown are means of five replicates, each composed of 30 seeds from each genotype. Statistically significant changes (Tukey-Kramer HSD test, *P* ≤0.05) between plants are identified by different letters.

Changes in protein accumulation and cellular ingredients could affect seed yield, seed weight, and germination rate. Since we did not find any significant changes in the yield between the different transgenic lines, the total weight of 100 seeds was determined for each set to reveal that SSE has a significantly higher level (6%), while A1 has a significantly lower weight of 23% compared to that detected in EV. The weights of the other lines did not change significantly compared to EV (**Figure 7A**).

The germination rate was determined by the emergence of radicals, and it was measured 1 and 2 days after the cold imbibition treatment for all of these genotypes. The results showed that there was no difference in germination rate for both progenies of crosses (A1S/A2S) compared to seeds expressing *AtD-CGS* and *SSA* alone, but all of these lines germinated better than EV and reached the maximum germination rate by the second day following imbibition (**Figure 7B**).

## Discussion

### Revealing the Factor/s Controlling the Accumulation of Protein-Bound Methionine in *A. thaliana* Seeds

The low levels of protein-bound methionine in cereals and legume crops limit the nutritional quality of seeds (Galili and Amir, 2013); therefore, an important biotechnological goal is to find approaches for increasing its levels. Generally, the low level of protein-bound methionine in seeds can result mainly from the low content of free methionine that restricts the synthesis of proteins and their accumulation, or from the number of methionine residues inside the endogenous seed-storage proteins [reviewed by Tabe et al. (1993); Galili and Amir (2013), and Amir et al. (2019)]. The previous observation that protein-bound methionine content increased by 65% in the water-soluble protein fraction of SSE suggested that higher levels of free methionine limit methionine accumulation in *A. thaliana* seeds (Cohen et al., 2014). The higher methionine content also affects the levels of proteins in the water-soluble fraction, which increased significantly by 10% in SSE seeds (Cohen et al., 2014).

However, the results of this study indicate that the level of protein-bound methionine in *A. thaliana* seeds is mainly limited by the methionine residues within the endogenous seed proteins. This suggestion is based on the following findings obtained from previous studies and the current study: i) Seed-specific expression of *AtD-CGS* in the first generation of SSE seeds, which led to a high level of free methionine (six-fold), elevated the level of protein-bound methionine in the water-soluble protein fraction only by 1.65-fold compared to EV (Cohen et al., 2014), while the level of protein-bound methionine in this fraction in *A. thaliana* seeds expressing the *SSA* alone (A1 and A2) increased significantly by 5.3- and 10.5-fold, respectively, compared to EV. This suggests that the *A. thaliana* seeds have enough free methionine content to support this accumulation; (ii) in seeds of SSE, the level of free methionine remained high (sixfold; Cohen et al., 2014), proposing that the capacity of methionine residues in the endogenous proteins came to saturation; iii) the increased level of the water-soluble protein fraction in seeds expressing SSA, A1, and A2 (24% and 38%, respectively) compared to EV (**Figure 4**) also suggests that there is enough free methionine in *A. thaliana* that can support this elevation; and iv) differences were detected between A1 and A2 in their protein-bound methionine in the water-soluble fraction. Compared to A1, A2 showed an approximately twofold higher expression level of *SSA* at the transcript and protein levels, which was in accordance to the twofold higher level of protein-bound methionine in the water-soluble fraction and about twofold lower level in its free methionine (**Figure 5**). This observation strongly implies that if the expression level of SSA in A1 would increase further, the level of free methionine would still support the accumulation of SSA. We assume that in A1, the expression level of SSA triggered the synthesis or accumulation of free methionine (**Figure 5**), but since the expression level of SSA in this line did not reach that of A2 (**Figure 2**), the free methionine did not succeed to incorporating into the SSA protein and thus remained at a high level (**Figure 5A**). All of these lines of evidence support the assumption that the protein-bound methionine in the water-soluble fraction in *A. thaliana* seeds is limited mainly by methionine residues in the seed proteins. Despite these lines of evidence, it should be stated that the results and assumptions are based on only two transgenic lines expressing the SSA gene (A1 and A2) and two progenies from a single cross with SSE (A1S and A2S).

The higher level of protein-bound methionine that was detected in *A. thaliana* seeds expressing *SSA* (5.3- and 10.5-fold in A1 and A2, respectively) was higher than other transgenic seeds expressing the same gene. The level detected in grains of lupins was twofold (Tabe et al., 2010) and 90% (Molvig et al., 1997), in chickpea grains by 90% (Chiaiese et al., 2004), while in rice, it increased by 75% (Lee et al., 2003) or changed insignificantly (Hagan et al., 2003) compared to control seeds. The proteins of *A. thaliana* seeds most probably have the lowest endogenous methionine residues in their proteins. However, the differences in the results could also be explained by sulfur availability, as was previously shown, for example, in chickpea and lupin growing with low and regular levels of sulfur (Tabe and Droux, 2001; Tabe and Droux, 2002; Chiaiese et al., 2004). Yet, the selected gene and the promoter also played a major role in methionine accumulation in seeds. This, for example, was shown in rice when the sesame 2S albumin was expressed under the control of a rice seed-specific glutelin promoter, in which the level of protein-bound methionine increased by 75% (Lee et al., 2003), but when *SSA* was expressed under the wheat’s high molecular weight of glutenin promoter, no significant changes were detected compared to the control seeds (Hagan et al., 2003).

In addition to the content of the methionine residues inside proteins, the level of free methionine could also be a limiting factor for the content of protein-bound methionine in seeds. To test this possibility, the levels of free methionine increased by the seed-specific expression of genes related to methionine biosynthesis pathway. The expression of these genes led to a range between non-significant elevations in azuki beans expressing *mto1-AtCGS* (Hanafy et al., 2013) up to a fourfold increase in maize expressing serine acetyltransferase (Xiang et al., 2018). The level of free methionine in these seeds increased twofold in azuki bean seeds, while in maize, it increased fourfold compared to the control seeds (Hanafy et al., 2013; Xiang et al., 2018). In between this range, one can find tobacco seeds expressing the bacterial aspartate kinas or *AtCGS* whose protein-bound methionine increased by 6.5% or 97%, with threefold and twofold elevations in their free methionine (Karchi et al., 1993; Matityahu et al., 2013), and also two soybean strains expressing the *AtD-CGS* having 2.3- to 3-fold elevations in their protein-bound methionine and 91% and 3.2-fold elevation in their free methionine (Song et al., 2013; Cohen et al., 2016b). In the current study, expression of *AtD-CGS* in *A. thaliana* SSE seeds increased free methionine 1.8-fold and protein-bound methionine 2-fold compared to EV. These results, as well as others [reviewed by Galili and Amir (2013) and Amir et al. (2019)], suggest that each genotype has a different methionine synthesis ability and/or different methionine residues in their endogenous proteins. Some genotypes are limited mainly by the methionine residues in their proteins (as in *A. thaliana*, azuki beans) and others by their free methionine content (as in tobacco). The results also suggest that many different factors could be at play in regulating the protein-bound methionine in seeds, and thus, each approach should be empirically tested separately in the seeds of each genotype.

### Revealing Whether the Combination of the Two Approaches (“Push–Pull”) Increases the Level of Protein-Bound Methionine in Transgenic Seeds

In the current study, it was detected that A2 expressing *SSA* alone and progenies of the “push–pull” approach (A1S, A2S) showed similar high levels of protein-bound methionine in the water-soluble protein fraction, which were 10.4- to 11.5-fold higher than EV (**Figure 5A**). As mentioned above, this suggests that A2 reached the maximum capacity of methionine residues for methionine incorporation. The observation that a higher production of free methionine did not affect the level of protein-bound methionine was also reported in lupin seeds expressing serine acetyltransferase (in the sulfur assimilation pathway; **Figure 1**) together with the 2S albumin of Brazil nut. The level of protein-bound methionine in these seeds did not increase beyond the levels detected in their parents (Tabe et al., 2010). However, this is unlike the results found in narbon bean seeds, which expressed the feedback-insensitive bacterial aspartate kinase and the 2S albumin of Brazil nut (Demidov et al., 2003). In these seeds, the level of methionine increased up to 2.4-fold compared to control seeds, and they also had more methionine than their parents: 10–12% more than those expressing the aspartate kinase and 80% more than those expressing Brazil nut (Demidov et al., 2003).

This combined approach was also tested in vegetative tissues, showing that unlike tobacco plants over-expressing *AtD-CGS* and 15 kDa zein in which the level of zein did not alter compared to plants over-expressing only 15 kDa zein (Golan et al., 2005), the level of this latter protein increased significantly in alfalfa transgenic plants expressing the same genes (Bagga et al., 2005; Golan et al., 2005). This elevation was concurrent with a reduction in the level of free methionine when compared to plants expressing *AtD-CGS* alone, implying that more free methionine was incorporated into the zein in the crossed alfalfa plants (Bagga et al., 2005; Golan et al., 2005). This strategy using the same enzymes was also tested in transgenic potatoes, leading to a sixfold increase in free and protein-bound methionine in the zein-containing protein fraction of the transgenic tubers (Dancs et al., 2008). The results from different studies trying the “push–pull” approach suggest that the successes depend on the genotype and the selection of genes. Remarkably, the results are promising and suggest that in certain seeds, this approach can lead to higher levels of protein-bound methionine, which is a very important aspect regarding the nutritional quality of seeds.

### The Effect of High Protein-Bound Methionine on the Levels of Storage Proteins

It was previously detected that a high expression level of *AtD-CGS* and thus a high level of methionine in SSE seeds increased the content of total proteins (Cohen et al., 2014; Cohen et al., 2016a). A high content of protein was also reported in the seeds of two transgenic lines of soybean (Song et al., 2013; Cohen et al., 2016b) and in tobacco seeds expressing the same *AtD-CGS* (Matityahu et al., 2013). These results suggest that the level of free methionine limits protein accumulation. These elevations are most probably also related to the high levels of other free amino acids whose levels were found to increase in these transgenic lines. High elevation of amino acids was also detected in the leaves of two *A. thaliana* lines having 1.75- and 2.25-fold higher contents of free methionine (Kim and Leustek, 2000). At least in the transgenic seeds, the total protein-bound amino acids also increased (Song et al., 2013; Cohen et al., 2014; Cohen et al., 2016b).

The high content of water-soluble proteins in SSE seeds (**Figure 4**; Cohen et al., 2014) suggests that high levels of free methionine change the levels of several storage proteins. It was indeed previously shown using 2D-gels and MALDI-MS that the accumulation of major storage proteins 12S-globulins and 2S-albumins changed in SSE (obtained from the first generation of SSE). It was also revealed that these changes were induced only from a certain threshold of free methionine, regardless of their methionine residue contents (Cohen et al., 2016a). The ability of the protein profile of seeds to change was also observed in A1S/A2S when the water-soluble proteins fraction was detected. The higher level of protein in this fraction in A1S compared to A1 (**Figure 3**) suggests that significant effects occurred when the level of methionine increased. However, this elevation in A1S was not reflected in the levels of water-soluble protein fractions (**Figure 4**), which were similar to those detected in SSE. We assume that this happens because the levels of some of the other storage proteins increased in A1S (in addition to SSA) as it occurred in SSE. In addition, the observation that the level of the water-soluble protein fraction obtained in SSE was similar to that measured in A2S suggests that SSA accumulation was at the expense of other endogenous proteins in the seeds. The results also propose that the seeds have a compensatory response to maintain stationary protein levels. This response might occur through the activities of promoters. In the current study, the heterologous genes are driven by the vicilin (SSA) and phaseolin (SSE) promoters, both of which drive genes encoding seed storage proteins, and thus may inherently be subject to the influences of amino acid and protein contents. However, these assumptions require further study. In any case, these assumptions are in accordance with other studies showing that while heterologous methionine-rich storage proteins accumulate in transgenic seeds, the level of other endogenous proteins had decreased. These include observations in rice, maize, soybean, and lupin seeds expressing methionine-rich storage proteins (SSA, 10 kDa zein, or the 2S Brazil nut) whereby the seeds did not show an increase in the level of protein-bound methionine, while instead the endogenous seed protein composition was markedly altered in such a way that the sulfur-poor proteins became over-abundant, while endogenous sulfur-rich proteins were down-regulated (e.g., Jung, 1997; Tabe and Droux, 2002; Hagan et al., 2003). These fluctuations might result due to alternation at the transcript expression level of seed-storage proteins. It was previously reported that high expression levels of SSA and serine acetyltransferase that increased the level of cysteine alter the transcript expression levels of several lupin seed storage proteins (Tabe and Droux, 2002). In addition, the transcript expression levels of sulfur-poor proteins were up-regulated when the level of *O*-acetyl serine (metabolite from the sulfur assimilation pathway; **Figure 1**) increased, and down-regulated when the level of free methionine increased (Hirai et al., 2002; Chiaiese et al., 2004), suggesting a direct effect of these metabolites on the transcript level of sulfur-poor storage proteins. The studies showing that the level of protein-bound methionine does not increase when the level of methionine-rich storage protein is expressed, together with studies showing that the levels of sulfur-containing metabolites decreased in several transgenic seeds (Molvig et al., 1997; Muntz et al., 1998), suggest that the level of free methionine limits the accumulation of proteins, and thus, they suggest examining the “push–pull” approach.

### Transgenic Seeds With Lower Free Methionine Content Exhibited Less Accumulation of Stress-Associated Metabolites Compared to Seeds With High Levels of Free Methionine

The fourth goal of the current study was to gain more evidence of the relationship between a high level of free methionine and the accumulation of stress-related metabolites. In a prior study, it was reported that SSE seeds with high levels of free methionine are accompanied by higher levels of other free amino acids (62% more than in EV) (Cohen et al., 2014). Such elevations were also reported in transgenic *A. thaliana* seeds expressing *RNAi-AtCGS* that have a high level of free methionine (Cohen and Amir, 2017; Cohen et al., 2017). In these transgenic seeds, the levels of other stress-related metabolites were also increased significantly, and the expression levels of genes related to drought and osmotic stresses were significantly up-regulated (Cohen et al., 2014; Cohen and Amir, 2017). The reasons for these elevations are not yet clear, but the positive correlation between higher levels of free methionine to higher levels of other amino acids and stress metabolites was also shown in two varieties of soybean seeds expressing *AtD-CGS* (Song et al., 2013; Cohen et al., 2016b), in tobacco leaves over-expressing *AtCGS* (Hacham et al., 2017), and *A. thaliana* leaves having higher levels of methionine (Kim and Leustek, 2000).

We hypothesized that if high levels of free methionine cause this effect in *A. thaliana* seeds, then a reduction in its level due to higher incorporation of methionine into seed proteins should reduce the levels of these metabolites. Indeed, the metabolic profiling that was conducted on the different sets of seeds shows that compared to SSE, the three sets of seeds, A2, A1S, and A2S, which show lower levels of free methionine (similar to EV), have lower levels of 29 out of 56 metabolites. These reduced metabolites are considered to be associated with stress responses such as sugars, free amino acids, organic acids, polyols, and several other metabolites (Ingram and Bartels, 1996; Angelovici et al., 2009; ElSayed et al., 2014). Moreover, the level of catechin, which is associated with abiotic stress responses, was reduced significantly in A2, A1S, and A2S compared to SSE (**Supplementary Table S2**).

A1 seeds expressing *SSA* alone also have a significant higher level of free methionine. Twenty metabolites increased significantly in this line compared to A2, A1S, A2S, and EV, most of which were related to abiotic stress (**Supplementary Table S2**; **Table 2**) (Ingram and Bartels, 1996). In addition, all of the top 25 metabolites that were found to be correlated with methionine were positively correlated (**Supplementary Figure S3**), and only 13 out of 56 metabolites had a negative correlation (**Supplementary Figure S4**). High positive correlation between high elevation of 21 out of 66 metabolites with methionine was also detected in soybean seeds (Cohen et al., 2016b). Taken together, our findings considerably strengthen the assumption that high levels of free methionine lead to higher accumulation of metabolites related to stress responses. The reason for this connection is yet unknown and definitely requires further studies.

### The Effect of Various Manipulations on Seed Weight and Germination Efficiency

The seeds of progenies of the crosses A1S and A2S, as well as SSE, exhibited significantly higher levels of water-soluble protein fraction compared to EV (**Figure 4**), and they also had similar seed dry weights (**Figure 7A**). This indicated that higher levels of water-soluble protein and protein-bound methionine in these seeds contributed to the significant increase in seed weight. This is in accordance with the results obtained for the seeds of A1, which exhibit relatively lower water-soluble protein content, and the protein-bound methionine was lower compared to A1S, A2S, and SSE.

The higher germination rate of SSE, A1, A2, A1S, and A2S seeds compared to EV suggests that the higher levels of proteins and other soluble metabolites contribute to improved germination efficiency (**Figure 7B**). This is unlike the first generation of SSE that shows the same germinate rate as the control seeds. However, SSE seeds show better germination rates under osmotic and salt stresses than WT seeds (Cohen et al., 2014). The results also differ from tobacco seeds expressing *AtD-CGS*, whose germination rates were significantly lower than that of the WT (Matityahu et al., 2013). Taken together, our results imply that the manipulation leads to a high level of proteins and to a better germination rate.

## Conclusion

Four objectives were addressed in this study; all were related to methionine metabolism in seeds. The first was to obtain more knowledge about the factor/s limiting the accumulation of protein-bound methionine content in *A. thaliana* seeds that are used as a model plant for *Brassicaceae*, as well as for other plants. The second goal was to reveal if the level of methionine could be increased further by using the “push–pull” approach. The third goal was to study how high levels of SSA and methionine affect the level of total water-soluble proteins in seeds. The fourth goal was to strengthen the assumption that an association exists between higher levels of free methionine and the accumulation of stress-related metabolites in seeds.

The results showed that free methionine levels in Arabidopsis seeds can increase the protein-bound methionine in seeds as shown in the seeds of SSE. However, expressing *SSA* led to a significantly higher level of protein-bound methionine compared to SSE (about 5-fold), suggesting that the content of methionine residues in the storage protein is the main limiting factor of methionine accumulation in Arabidopsis seeds as shown in A2 plants. Moreover, the “push–pull” approach obtained by crossing plants expressing seed-specific *AtD-CGS* and those expressing the *SSA* did not lead to significantly higher levels of protein-bound methionine beyond the level obtained by A2 expressing the *SSA* alone. This indicates that the methionine residues in A2 line reach a maximum threshold for the incorporation of free methionine. More than that, it also proposed that expression levels of SSA in A1 increased the methionine biosynthesis pathway and elevated the free methionine content. The results achieved in A1 and A1S suggested that the “push–pull” strategy should be tested in other seeds, since its applicability could lead to methionine improvement that could increase the nutritional value of these seeds. The results obtained from the water-soluble proteins measurements showed that their levels were similar in SSE and in the progenies of the crosses (A1S, A2S), suggesting that the accumulation of *SSA* in seeds was at the expense of other endogenous proteins that accumulate in SSE. The results of this study also suggested a link between higher free methionine content and accumulation of other amino acids, sugars, polyols, and metabolites that accumulate in plants during stress. This link is interesting but the reason is still unknown and thus requires further studies.

## Data Availability Statement

All datasets presented in this study are included in the article/**Supplementary Material**.

## Author Contributions

YH and RA: Experimental design. AG, DS, and YH: Conducted the experiments. AG, DS, and YH: Data analysis. AG, YH, and RA: Manuscript preparation. All authors have read and approved the manuscript.

## Funding

This work was supported by grants from the Israeli Science Foundation (grant no. 1004/15).

## Conflict of Interest

The authors declare that the research was conducted in the absence of any commercial or financial relationships that could be construed as a potential conflict of interest.
