# Supplementary material for: The Level of Methionine Residues in Storage Proteins Is the Main Limiting Factor of Protein-Bound-Methionine Accumulation in Arabidopsis Seeds
Source: Front Plant Sci. 2020 Aug 5;11:1136. doi: 10.3389/fpls.2020.01136 (PMC7419676; doi:10.3389/fpls.2020.01136)
Supplement: Supplementary file 2 [file DataSheet_2.docx]

**Supplementary Text. The transcript expression level of *SSA* and *AtD-CGS* in the transgenic lines**

To assess how higher expression levels of both genes (*SSA* and *AtD-CGS*) affect their transcript expression levels, quantitative real-time PCR (qRT-PCR) analyses were performed. The progenies of A1S showed a 25% higher expression level of *SSA*  compared to its parent, A1, while A2S showed no significant differences compared to its parent, A2 (Supplementary Figure S1). Next, the expression levels of *AtD-CGS* were detected in the transgenic lines. The expression of *AtCGS* is known for its feedback regulation by increased SAM levels, the main catabolic product of methionine ([Chiba et al., 2003](#_ENREF_10)). The expression level of *AtD-CGS* in seeds of A2S was similar to that detected in SSE, while that measured in A1S was unexpectedly significantly lower by 1.8-fold (Supplementary Figure S1). The expression level of total *AtCGS* (endogenous plus the transgene) was also measured in the parents and the progenies of these crosses. The transcript expression level of total *AtCGS* in SSE was 9.6-fold higher than EV, which was significantly lower than the value previously reported for the first generation of SSE (58-fold) ([Cohen et al., 2014](#_ENREF_14)). However, unexpectedly, the A1S and A2S (having this heterologous gene) showed a significant 2.3- and 2.75-fold down-regulation, respectively, of total *AtCGS* compared to SSE (Figure 2D). This result is unexpected since most of the free methionine in these seeds should be incorporated into proteins, and thus the expression level of *AtCGS* should increase. The reason for such a reduction is not yet clear. Nevertheless, the expression level of *AtCGS* showed a significant 4- and 3-fold increase in A1 and A2, respectively, compared to EV, suggesting that more free methionine was incorporated into *SSA*. The reduction of free methionine, lowering the feedback inhibition on the endogenous *AtCGS*, and thus enables the accumulation of *AtCGS*. Another explanation is that the high expression of *SSA* enhances the demand for soluble methionine, which up-regulates the expression of genes in the methionine biosynthesis pathway. Similar to the latter assumption, it was previously suggested that when *SSA* was expressed in lupin seeds, the flux through the biosynthetic pathways of methionine and cysteine increased in the transgenic seeds ([Tabe and Droux, 2002](#_ENREF_54)).
