# Supplementary material for: The Level of Methionine Residues in Storage Proteins Is the Main Limiting Factor of Protein-Bound-Methionine Accumulation in Arabidopsis Seeds
Source: Front Plant Sci. 2020 Aug 5;11:1136. doi: 10.3389/fpls.2020.01136 (PMC7419676; doi:10.3389/fpls.2020.01136)
Supplement: Supplementary file 3 [file Presentation_1.pptx]

## Slide 1
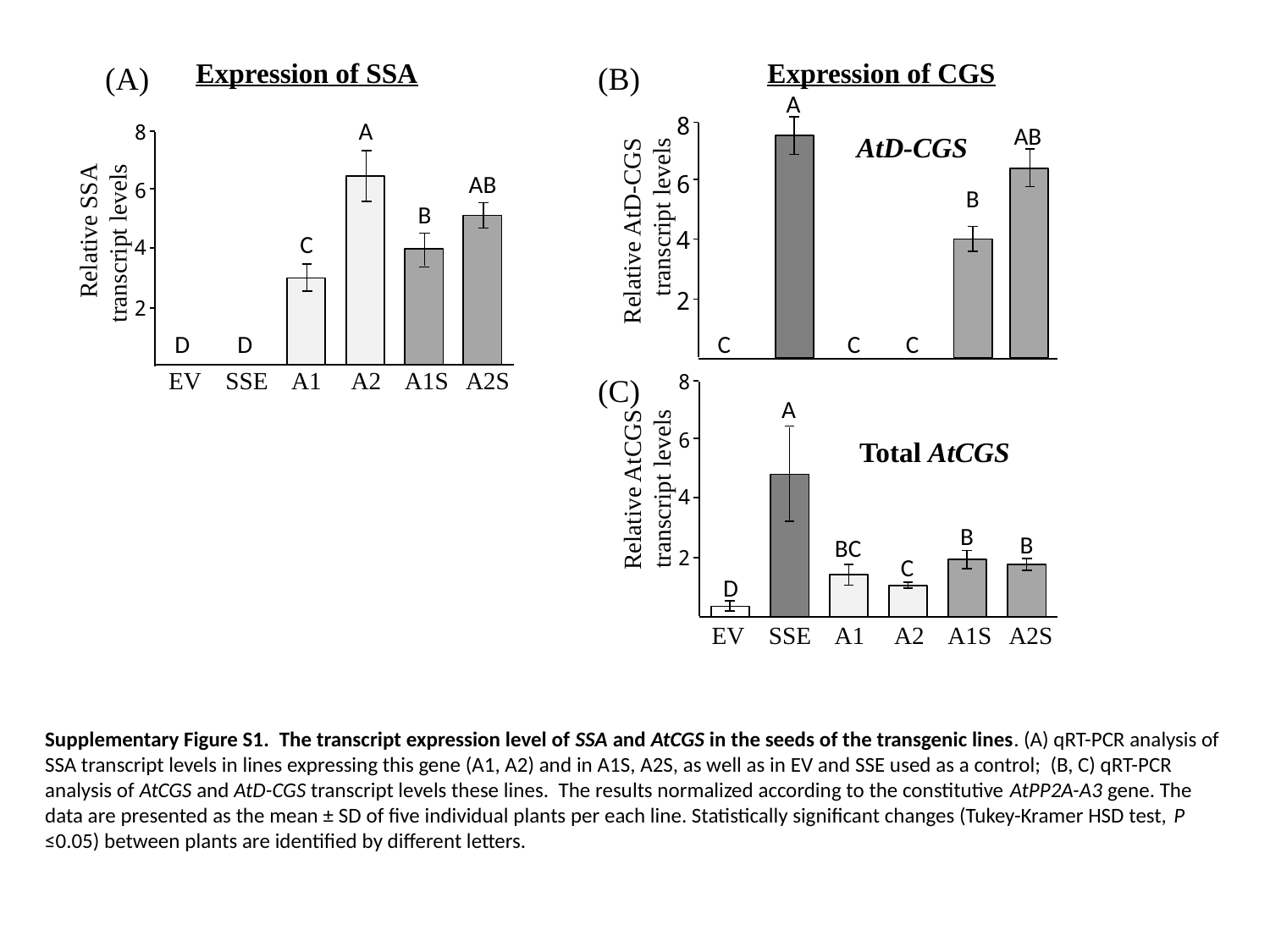

Expression of SSA
Expression of CGS
(A)
(B)
A
8
AB
AtD-CGS
6
B
Relative AtD-CGS
transcript levels
4
2
C
C
C
A
8
AB
6
B
Relative SSA
transcript levels
C
4
2
D
D
EV SSE A1 A2 A1S A2S
8
A
6
Total AtCGS
Relative AtCGS
transcript levels
4
B
B
BC
2
C
D
(C)
EV SSE A1 A2 A1S A2S
Supplementary Figure S1. The transcript expression level of SSA and AtCGS in the seeds of the transgenic lines. (A) qRT-PCR analysis of SSA transcript levels in lines expressing this gene (A1, A2) and in A1S, A2S, as well as in EV and SSE used as a control; (B, C) qRT-PCR analysis of AtCGS and AtD-CGS transcript levels these lines. The results normalized according to the constitutive AtPP2A-A3 gene. The data are presented as the mean ± SD of five individual plants per each line. Statistically significant changes (Tukey-Kramer HSD test, P ≤0.05) between plants are identified by different letters.

## Slide 2
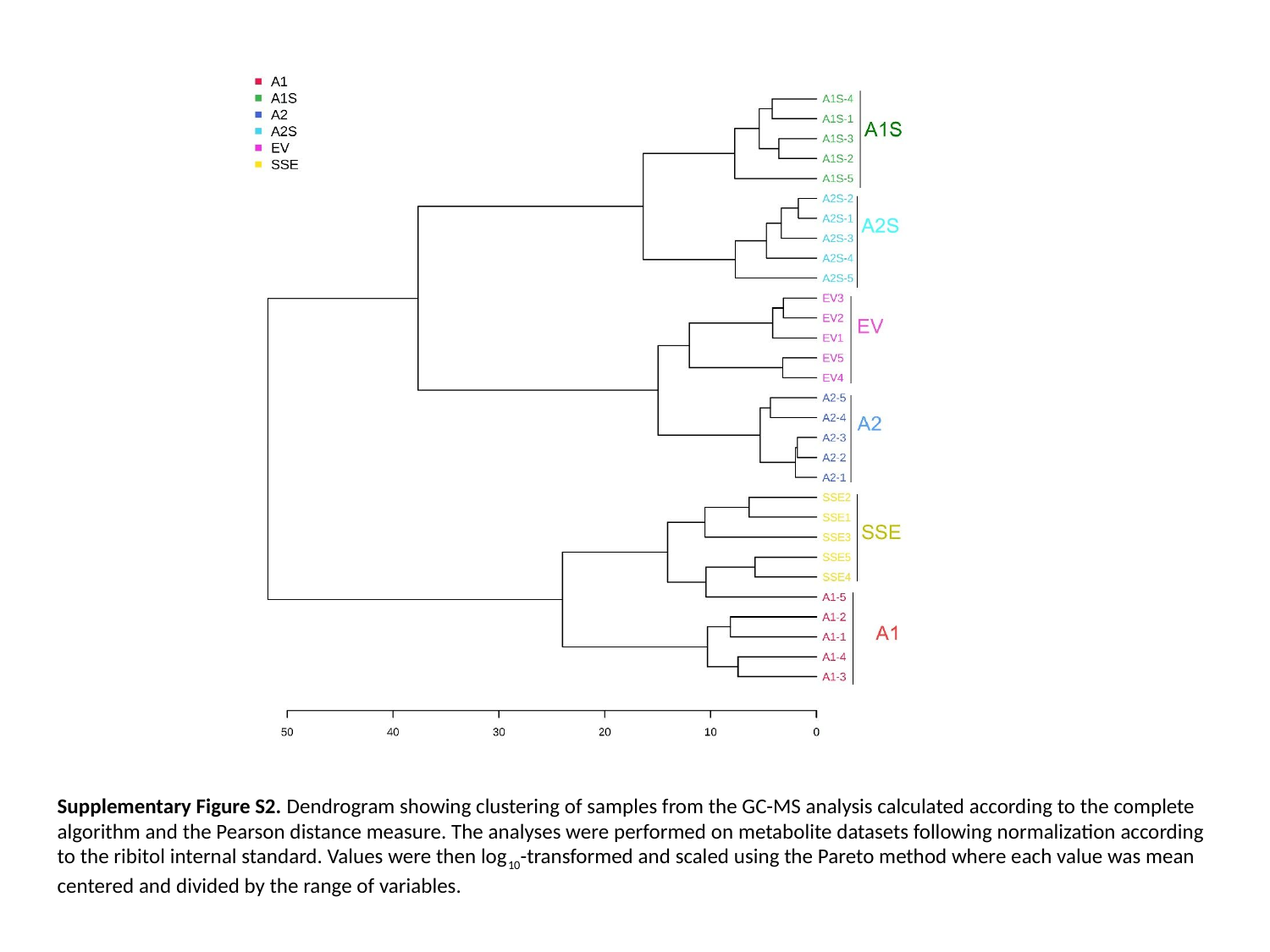

Supplementary Figure S2. Dendrogram showing clustering of samples from the GC-MS analysis calculated according to the complete algorithm and the Pearson distance measure. The analyses were performed on metabolite datasets following normalization according to the ribitol internal standard. Values were then log10-transformed and scaled using the Pareto method where each value was mean centered and divided by the range of variables.

## Slide 3
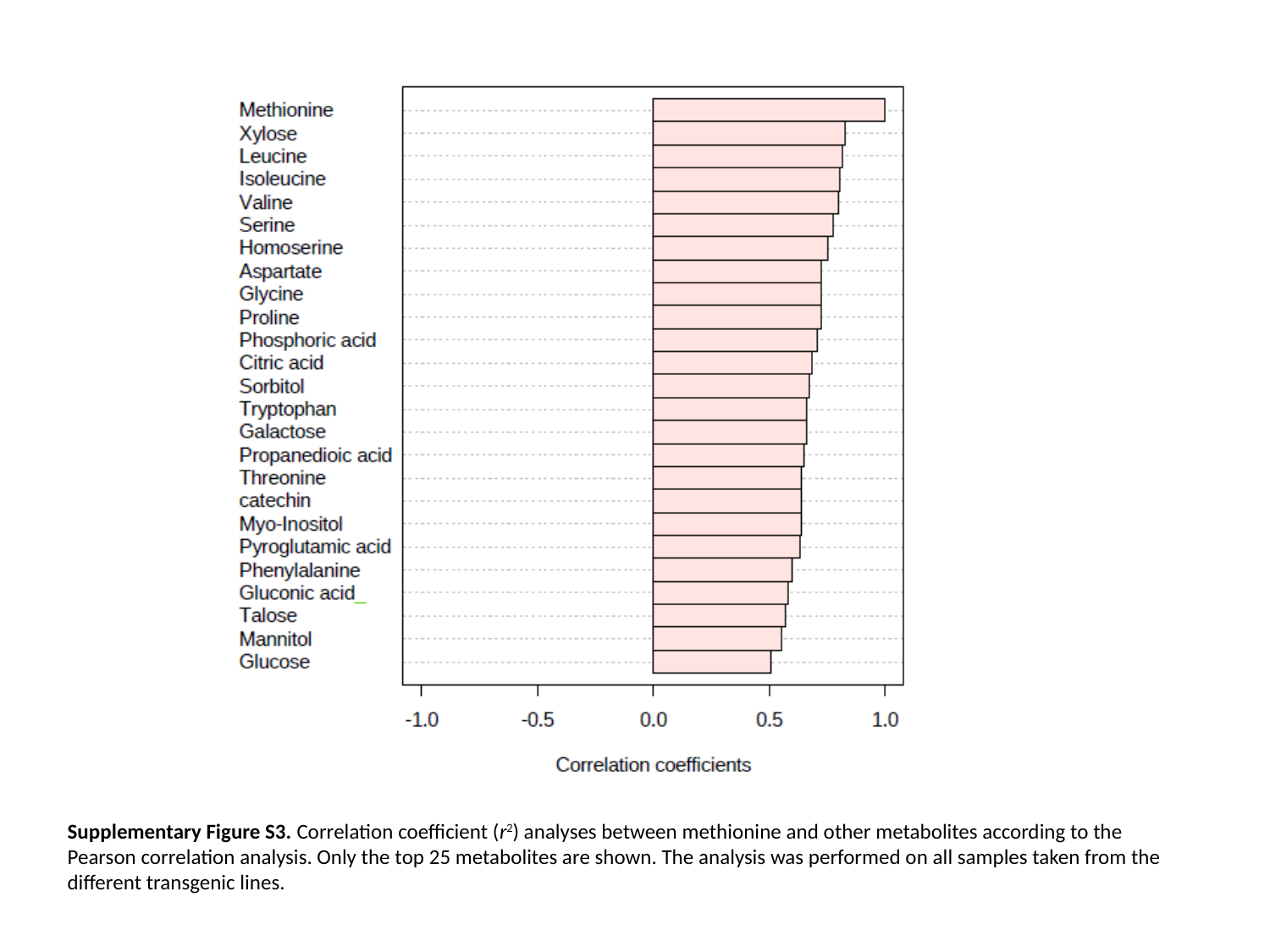

Supplementary Figure S3. Correlation coefficient (r2) analyses between methionine and other metabolites according to the Pearson correlation analysis. Only the top 25 metabolites are shown. The analysis was performed on all samples taken from the different transgenic lines.

## Slide 4
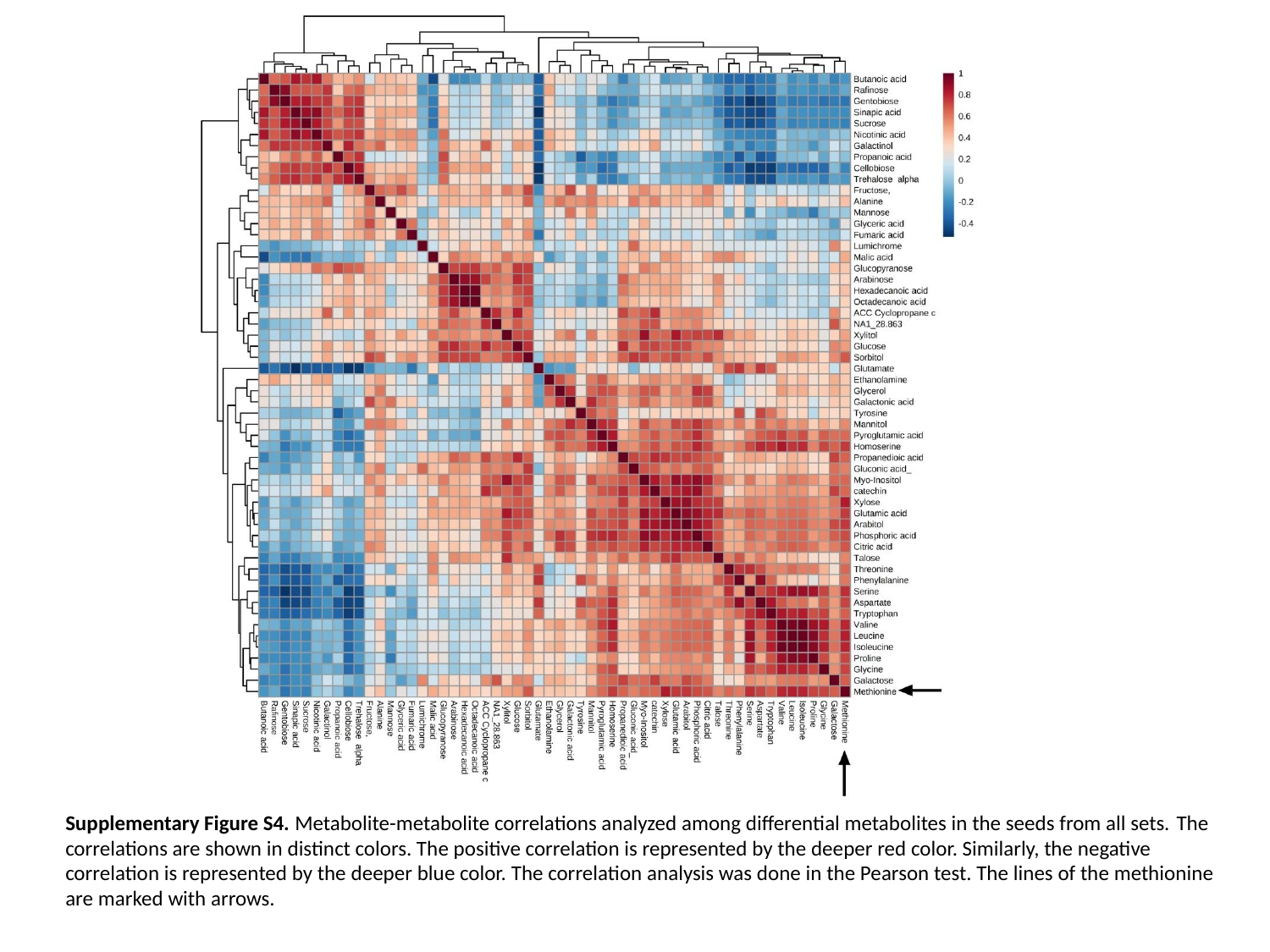

Supplementary Figure S4. Metabolite-metabolite correlations analyzed among differential metabolites in the seeds from all sets. The correlations are shown in distinct colors. The positive correlation is represented by the deeper red color. Similarly, the negative correlation is represented by the deeper blue color. The correlation analysis was done in the Pearson test. The lines of the methionine are marked with arrows.
